# MiR-19 regulates the proliferation and invasion of glioma by RUNX3 via β-catenin/Tcf-4 signaling

**DOI:** 10.18632/oncotarget.22720

**Published:** 2017-11-28

**Authors:** Jikui Sun, Zhifan Jia, Banban Li, Anling Zhang, Guangxiu Wang, Peiyu Pu, Zhijuan Chen, Zengguang Wang, Weidong Yang

**Affiliations:** ^1^ Department of Neurosurgery, Affiliated Hospital of Taishan Medical University, Life Science Research Center of Taishan Medical University, Taian, 271000, P.R. China; ^2^ Department of Neurosurgery, Tianjin Medical University General Hospital, Tianjin Neurological Institute, Key Laboratory of Post-trauma Neuro-repair and Regeneration in Central Nervous System, Ministry of Education, Tianjin Key Laboratory of Injuries, Variations and Regeneration of Nervous System, Tianjin, 300052, P.R. China; ^3^ Department of Hematopathology, Affiliated Taishan Hospital of Taishan Medical University, Taian, 271000, P.R. China

**Keywords:** miR-19, RUNX3, β-catenin, TCF4, glioma

## Abstract

Accumulating data demonstrates that the network dysregulation of microRNA-medicated target genes is involved in glioma. We have previously found miR-19a/b overexpression in glioma cell lines and specimens with various tumour grades. However, there was no report on the function and regulatory mechanism of miR-19a/b in glioma. In this study, based on our previous research data, we first determine the inverse relationship between miR-19 (miR-19a and miR-19b) and RUNX3 which is also identified the reduced expression in tumour tissues by real-time PCR and IHC. Luciferase reporter assay and western blot analysis revealed that RUNX3 was a direct target of miR-19. Down-regulation of miR-19 dramatically inhibited proliferation, invasion and induced the cell cycle G1 arrest and apoptosis, at least partly via the up-regulation of RUNX3. Furthermore, Mechanistic investigation indicated that knockdown of miR-19 repressed the β-catenin/TCF4 transcription activity. In conclusion, our study validates a pathogenetic role of miR-19 in glioma and establishes a potentially regulatory and signaling involving miR-19 /RUNX3/β-catenin, also suggesting miR-19 may be a candidate therapeutic target in glioma.

## INTRODUCTION

Glioblastoma is the most malignant and incurable primary brain tumour with poor prognosis despite progress in surgical techniques, radio-therapy and chemotherapy [1]. Glioblastomas are also characterized by multiple genetic alterations such as epidermal growth factor receptor (EGFR) amplification, PTEN mutations as well as various signaling pathways including PI3K/AKT/mTOR pathway, Wnt/β-catenin pathway and P53 signaling pathway [2, 3]. It is not sufficient to enable great progress in individualized and targeted therapy in spite of accumulating evidence of glioblastoma molecular pathology. Therefore, it is essential to further understand molecular mechanisms involved in the development and progression, providing insights into specific therapeutic strategies.

MicroRNAs are endogenously expressed regulatory noncoding RNAs that negatively regulate gene expression in post-transcription by directly binding to the 3′untranslated region (3′UTR) of target mRNAs [4]. Accumulating data indicate that dysregulation of microRNA activity has been shown to play crucial roles in tumour initiation and progression, including gliomagenesis [5, 6]. For instance, miR-21, the first verified the oncogenic microRNA in malignant gliomas, is involved in cell proliferation, apoptosis and invasiveness via targeting anti-tumour genes of P53,TGF-β and mitochondrial apoptotic pathways [7, 8]. Each microRNA can potentially regulate a considerable body of mRNA targets and function as oncogenes or tumour suppressors [9]. Our previous study demonstrated that miR-19 composed of miR-19a and miR-19b was overexpressed in glioma cell lines and tissue samples with different grades of malignancy, as verified by real-time PCR and in-situ hybridization [10]. However, its biological roles and the molecular mechanisms underlying gliomagenesis remain poorly understood.

RUNX3 gene is located in 1p36, a region of frequently genomic loss in a wide variety of human carcinomas, including glioblastoma [11, 12]. RUNX3 was reported to be downexpressed primary glioblastomas, and its overexpression resulted in significantly repressed cell invasion and migration abilities [13]. However, there was no report indicated the potentially regulatory and signaling pathway involving miR-19/RUNX3 in glioma.

In the present study, we first determine the inverse correlation between miR-19 and RUNX3. In addition, we also examined the reduced expression of RUNX3 in resected tissue specimens by real-time PCR and IHC, which is consistent with the previous results. Furthermore, our data demonstrated that RUNX3 is a direct target of miR-19 and suppression of miR-19 inhibited glioma cell proliferation, invasion and induced apoptosis at least partly through RUNX3, subsequently blocked the Wnt/β-catenin signaling pathway. Therefore, we explored the potential pathway involving miR-19/RUNX3/β-catenin trying to elucidate the related mechanism in glioma, also suggesting that miR-19 may be a possible diagnostic therapeutic approach that deserves further evaluation in GBM.

## RESULTS

### Analysis on expression of miR-19 and RUNX3 with the tumour grade

**Figure 1:**
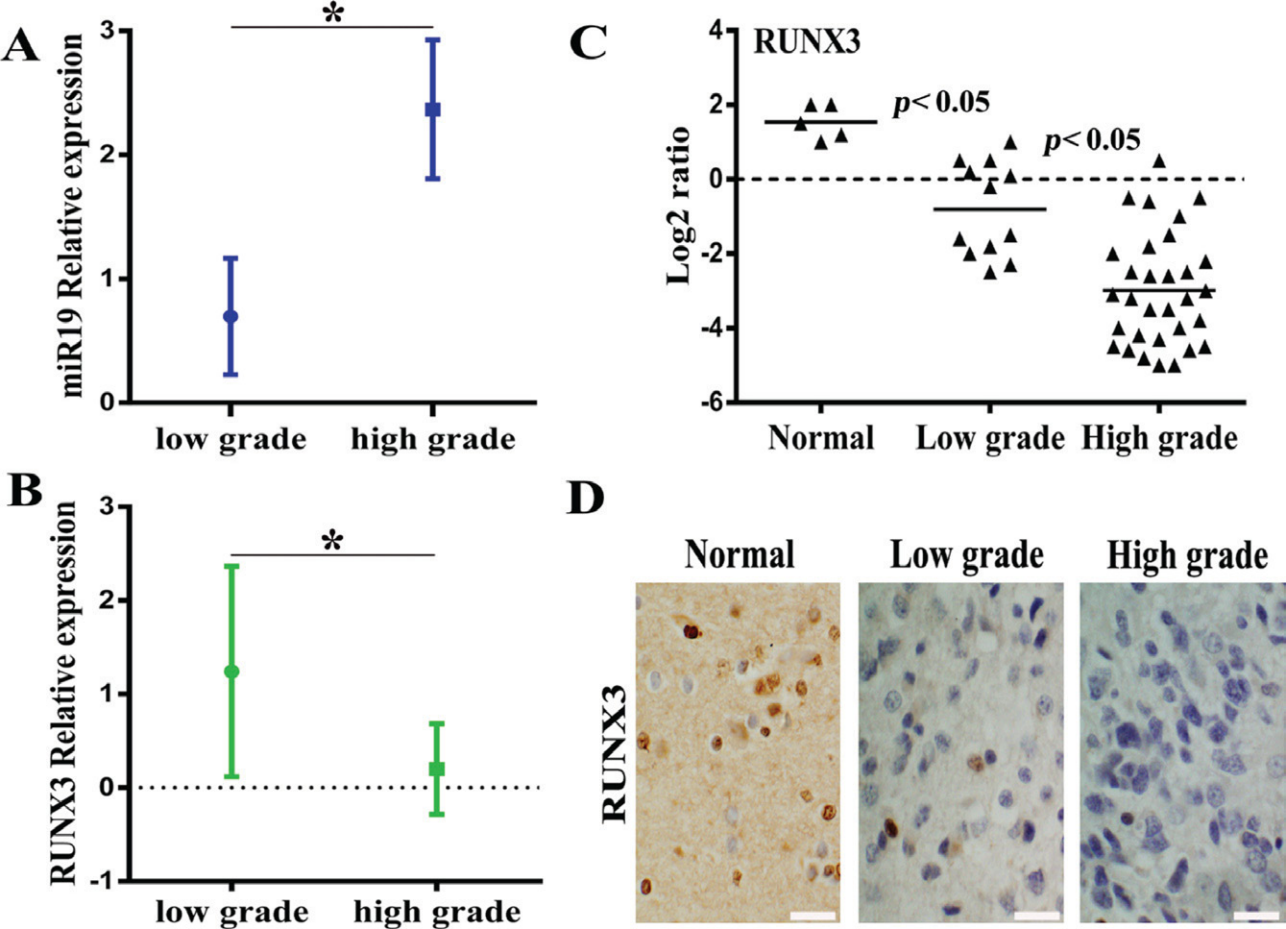
The inverse correlation between miR-19 and RUNX3 expression based on previous data of tissue chip (**A**) The relation between miR-19 expression and the tumor grade according to WHO grade. (**B**) The relation between RUNX3 expression and WHO tumor grade. (**C**) RUNX3 expression in different grades of glioma specimens and normal brain tissue by real-time PCR. (**D**)Expression of RUNX3 in resected gliomas specimen was detected by immunohistochemical staining assay (×200).* < 0.05.

Firstly, we analysize the relationship on expression of miR19 and RUNX3 with different tumour grades according to WHO classification scheme. Our previous study has demonstrated that miR-19 was overexpressed in malignant gliomas cell lines and tissue specimens and the results were published in Pathol. Oncol. Res. (2013). Moreover, our data also indicated that RUNX3 is frequently downregulated in gliomas cell lines and tissue specimens with qRT-PCR, western blot and immunohistochemical staining respectively (data not shown). Based on the recent results, association for expression of miR-19 and RUNX3 with the tumor grade was analyzed in Figure 1A and 1B, suggesting that miR-19 is positively correlated with the tumor grade while RUNX3 is highly expressed in normal brain tissue and negatively correlated with the tumor grade. To verify the expression of RUNX3 besides tissue microarray, we used Real-time PCR and Immunohistochemical staining to detect clinical 43 resected glioma tissue samples. Figure 1C and 1D indicated that RUNX3 is overexpressed remarkably in normal brain tissue compared with glioma specimens, especially high-grade gliomas.

### RUNX3 is a direct target gene of miR-19

**Figure 2:**
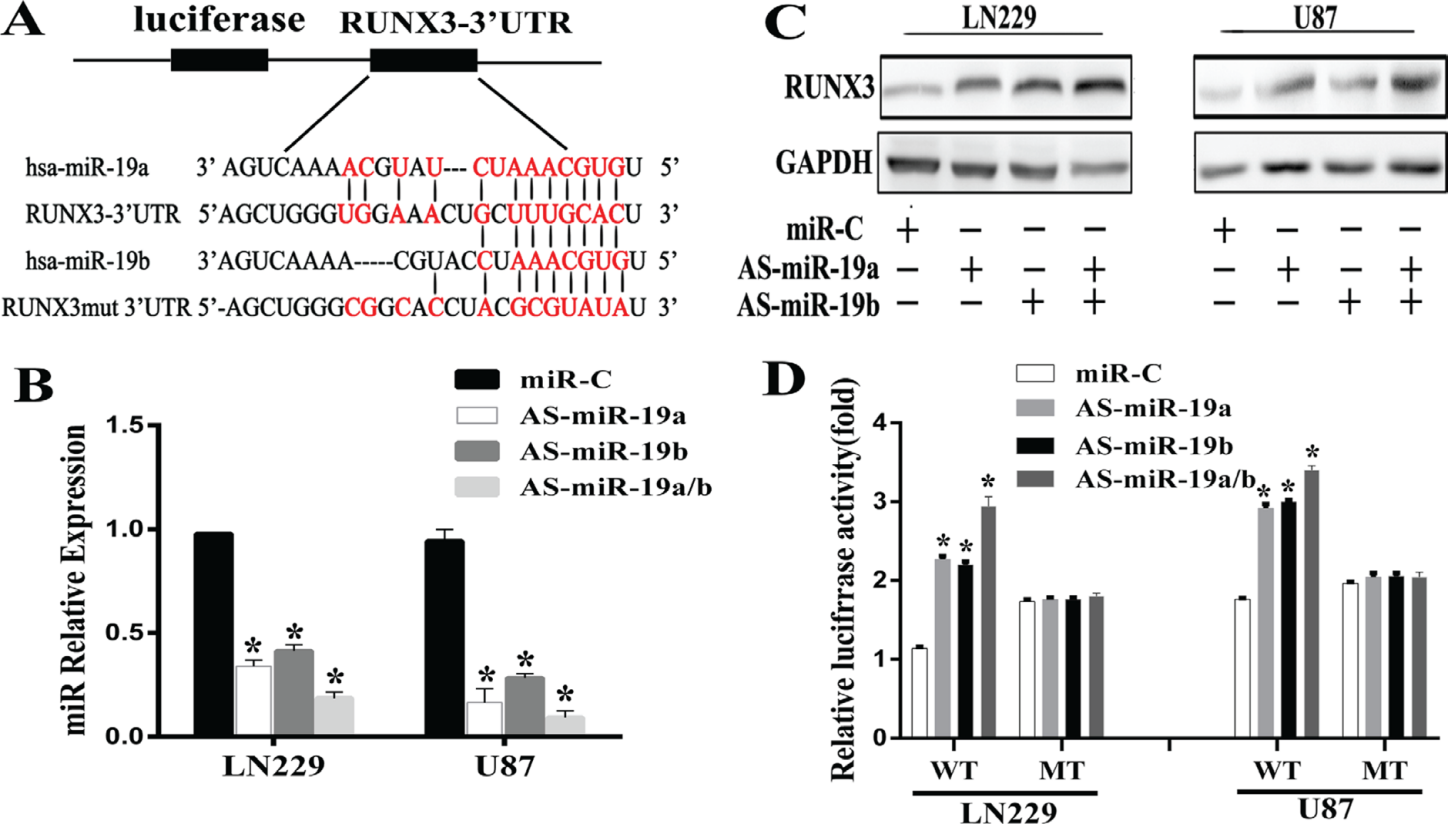
RUNX3 is a direct target of miR-19a/b in glioma cells (**A**) A schematic diagram of RUNX 3′UTR containing reporter contructs and seed sequence of miR-19a/b matched the 3′UTR of RUNX3. (**B**) AS-miR-19a/b blocked the miR-19a/b expression by real-time PCR 48h post transfection. (**C**) Western blot for RUNX3 expresion 48 h transfected with the miR-C/miR-19a/b inhibitors. GAPDH protein was served as endogenous normalizer. (**D**) Luciferase reporter assays in LN229 and U87 cells, following cotransfection with the wide-type or mutant 3′UTR of RUNX3 and AS-miR-19a/b, as indicated. The data represent the fold change in expression(mean ± SE) of three replicates. ^*^p < 0.05 compared with miR-C group.

To explore the detailed correlation between miR-19 and RUNX3, miRNA targets prediction database was searched through Targetscan, Pictar and miRanda. 3′UTR of RUNX3 was found to contain the highly conserved binding sites of miR-19a/b (Figure 2A). To test whether miR-19a/b regulates RUNX3 expression *in vitro*, we used AS-miR-19a/b (miR-19a/b antisense oligonucleotide) to reduce the miR19a/b expression level in gliomas LN229 and U87 cell lines. Our results demonstrated that AS-miR-19a/b could markedly decrease the miR-19a/b expression by real-time PCR, evident in cell lines co-transfected with AS-miR-19a and AS-miR-19b (Figure 2B). There was a decrease in the RUNX3 protein expression level 48h after transfection with AS-miR-19a/b (Figure 2C). To further verify RUNX3 is a direct target gene of miR-19a/b, we constructed the pEZX -RUNX3 plasmid containing 3′UTR of RUNX3 and conducted a reporter gene assay. As shown in Figure 2D, reporter assay revealed that the reduction of miR-19a/b led to a remarkable increase of luciferase activity in pEZX-WT-RUNX3 combined with AS-miR-19a/b transtracted cells, whereas no change of luciferase activity was found in the mutant pEZX-MUT-RUNX3 with miR-C (miR-control) transfected cells. These evidences indicated that miR-19a/b directly modulated RUNX3 expression by binding to 3′UTR of RUNX3.

### Down-regulation of miR-19 inhibits glioma cell proliferation and invasion and induces apoptosis partly dependent on the gene RUNX3 *in vitro*

**Figure 3:**
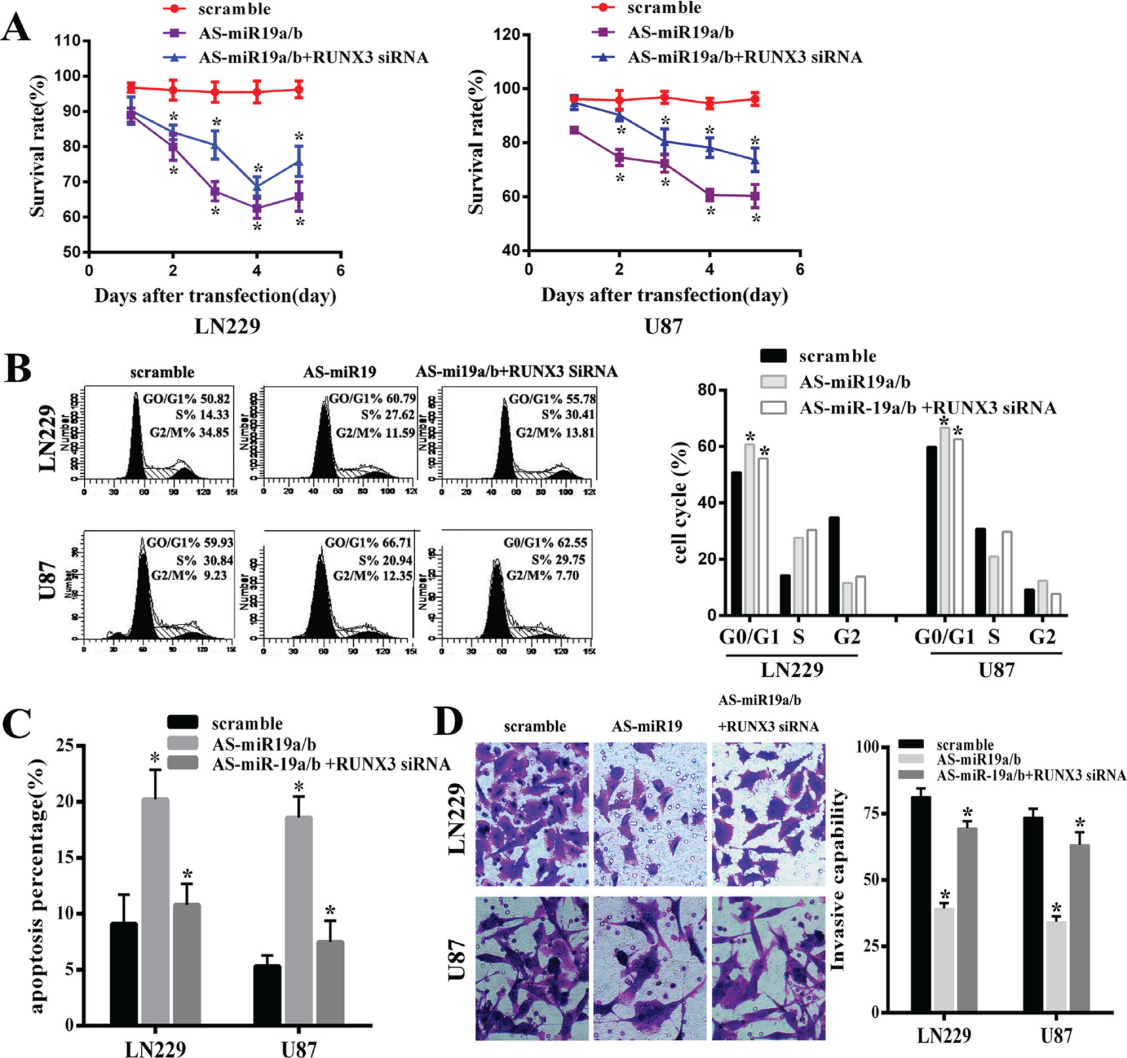
MiR-19a/b inhibition could repress proliferation, invasion, induce cell cycle G0/G1 arrest and promote cell apoptosis whereas these effects were partly reversed by downregulating the expression of the target gene RUNX3 in LN229 and U87 cell lines (**A**) MTT assay was used to examine the viability in cells transfected with AS-miR19a/b and co-transfected RUNX3 siRNA reducing the expression of RUNX3, compared with scramble group. (**B**) Flow cytometry data represented the cell cycle distribution.G0/G1 phase arrest was observed after transfected with AS-miR19a/b for 48 h while co-transfection of RUNX3 siRNA partly abrogated the effect of G0/G1 arrest by miR-19a/b inhibition compared with scramble group. (**C**) Apoptotic Index (AI) of scramble and transfected LN229 and U87 cells was analyzed through Annexin V staining. (**D**) Invasive capability was assessed by transwell assay indicating RUNX3 medicated AS-miR19a/b inhibitory apoptosis. The data are presented as the mean±standard deviation of triplicate samples of at least three independent experiments. ^*^p < 0.05.

Our results have demonstrated that AS-miR-19a/b could remarkably up-regulated expression of RUNX3. In order to explore the function of RUNX3 on glioma malignant phenotype by AS-miR-19a/b in LN229 and U87 cell lines, we used the small interfering RNAs to knockdown the RUNX3 level. A significant decrease in the cell viability was observed over time in glioma cells with low miR-19a/b expression compared with the cells that had no transfection of AS-miR-19 as observed with the MTT assay, moreover, knockdown of RUNX3 in cells transfected with AS-miR-19a/b partly abolished the effect on the cell prolification of AS-miR-19a/b (Figure 3A). Next, to analyze the mechanism of cell growth arrest caused by miR-19a/b inhibition, we assessed cell cycle distribution of glioma cells transfected with miR-19a/b inhibitor or control oligo as well as co-transfection between AS-miR19a/b and RUNX3 siRNA. MiR-19a/b inhibition caused cell cycle arrest in the G0/G1 phase, whereas the transfection of RUNX3 siRNA also abrogated the effect of AS-miR-19a/b on cell cycle arrest (Figure 3B). These observations suggest that miR-19a/b deletion suppresses the proliferation of glioma cells, partially dependent on the RUNX3 in LN229 and U87cell lines.

We further performed the invasive ability examination and apoptosis detection in LN229 and U87 cells transfected with AS-miR-19a/b and co-transfected with RUNX3 siRNA using transwell assays and flow cytometric analysis of Annexin-V-PI staining respectively. In both cell lines, the average apoptotic cell fractions (Early apoptotic + Apoptotic) were significantly increased with miR-19a/b inhibition compared to the scramble while the invasive ability of tumor cells markedly decreased after transfection of AS-miR-19a/b. Consistent with the results of proliferation, the inhibitory on invasion and apoptosis induction caused by AS-miR-19a/b were partly weakened obviously via co-transfection of RUNX3 siRNA which down-regulated the expression of RUNX3 (Figure 3C, 3D).

### RUNX3 partly restored phenotype effect of miR-19a/b repression in glioma cells

**Figure 4:**
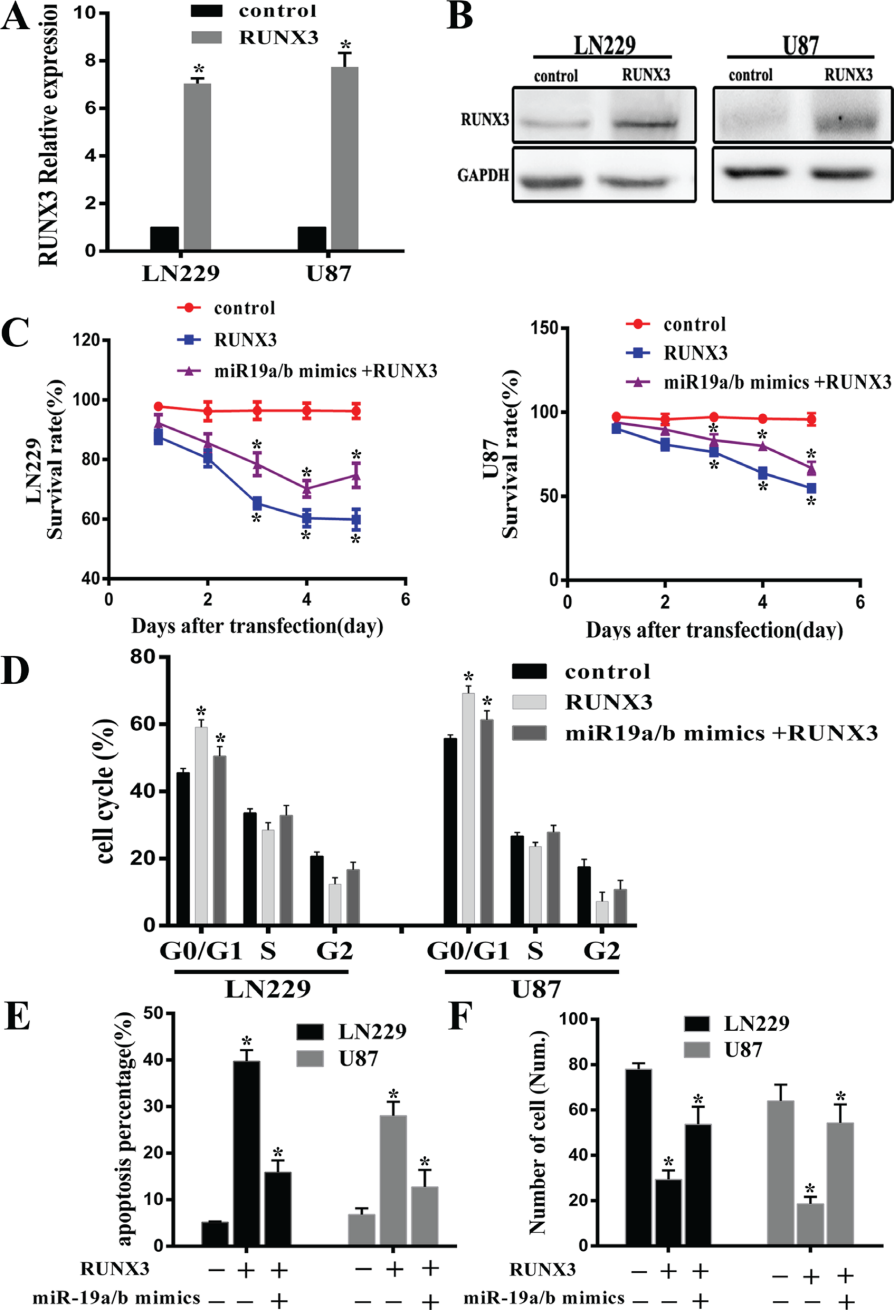
RUNX3 restoration partly reverses the tumorigenic effects of miR-19a/b Decreased proliferation and invasion, increased apoptosis as well as cell cycle G0/G1 arrest were observed in cells transfected RUNX3 recombinant advirus compared with control group. (**A**, **B**) RUNX3 mRNA and protein expression levels in U87 and LN229 cells transfected with RUNX3 recombinant advirus were assessed by real-time PCR and Western blot. (**C**–**F**) Representative cartogram showing MTT examination, cell cycle distribution, apoptosis and transwell assay in LN229 and U87 cells treated with RUNX3 upregulation and Co-transfection combined with miR-19a/b mimics. The data indicates restoration of RUNX3 expression counteracts the impact of miR19a/b in LN229 and U87cells. ^*^p < 0.05.

To further explore RUNX3 antitumor effect on miR-19a/b medicated cell biology, we examined the proliferation, cell cycle distribution, apoptosis and invasiveness in LN229 and U87 cells treated with RUNX3 recombinant adenovirus (rAd-RUNX3) and miR-19a/b mimics combined with rAd-RUNX3. Real-time PCR and western blot assay verified overexpression of RUNX3 after transfected rAd-RUNX3 (Figure 4A, 4B). Consistent with previous results, proliferation, cell cycle distribution, transwell and apoptosis assay revealed that RUNX3 restoration established a remarkable antitumor effect similar to phenotype observed upon miR-19a/b inhibition in LN229 and U87 cell lines. However, co-transfection of miR-19a/b mimics and RUNX3 in cells partly reversed the antitumour effects induced by RUNX3 (Figure 4C–4F). These results suggest the carcinogenesis effect of miR-19a/b in part facilitated by RUNX3 down-regulation.

### MiR-19a/b abrogation represses the β-catenin/Tcf-4 signaling pathway

**Figure 5:**
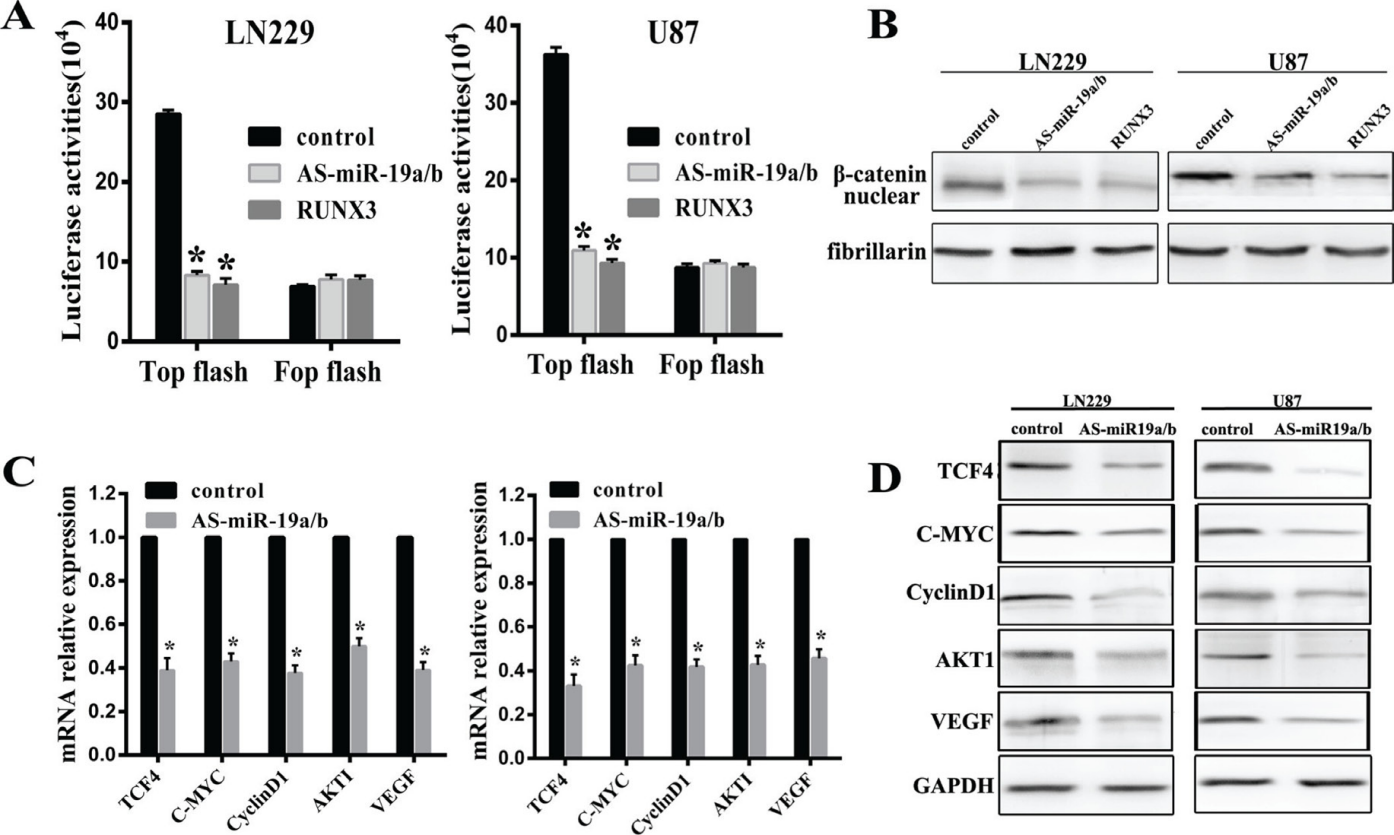
MiR-19a/b abrogation represses the β-catenin/Tcf-4 transcriptional activity (**A**) LN229 and U87 cells were co-transfected with Top/Fop and miR-19a/b inhibitors or RUNX3, after which luciferase reporter assays were performed. (**B**) Western blot detection of β-catenin 48 h following transfection with AS-miR19a/b and RUNX3 in both cell lines. (**C**, **D**) Real-time PCR and western blot detection of TCF4, C-MYC, CyclinD1, AKT1 and VEGF 48 h following transfection of LN229 and U87 cells with AS-miR19a/b. ^*^p < 0.05 compared with control group.

Our experimental data has established that RUNX3 could inhibit Wnt/β-catenin signaling pathway which has not been published. Combined with RUNX3 regulation by miR-19a/b, we put on hypothesis that miR-19a/b could regulate Wnt/β-catenin pathway. In order to test it, Top/Fop flash luciferase assay was utilized in cells in which miR-19a/b deletion or RUNX3 expression restoration. In LN229 and U87 cells, AS-miR-19a/b or RUNX3 reduced TOP with no obvious change in Fop flash luciferase activities (Figure 5A). In addition, western blot assay demonstrated that depletion of miR-19a/b or overexpression of RUNX3 decreased the expression of β-catenin in nucleas (Figure 5B).

Confirming the essential role of the β-catenin/Tcf-4 pathway as mediators of miR-19a/b, real-time PCR and western blot assay indicated reduced relative expression of TCF4, CyclinD1, C-MYC, AKT1 and VEGF in cells with low miR-19a/b expression (Figure 5C, 5D). These data suggest miR-19a/b regulate β-catenin/Tcf-4 transcriptional activities via RUNX3, at least in LN229 and U87 cells.

### AS-miR-19a/b inhibits tumor growth *in vivo*

**Figure 6:**
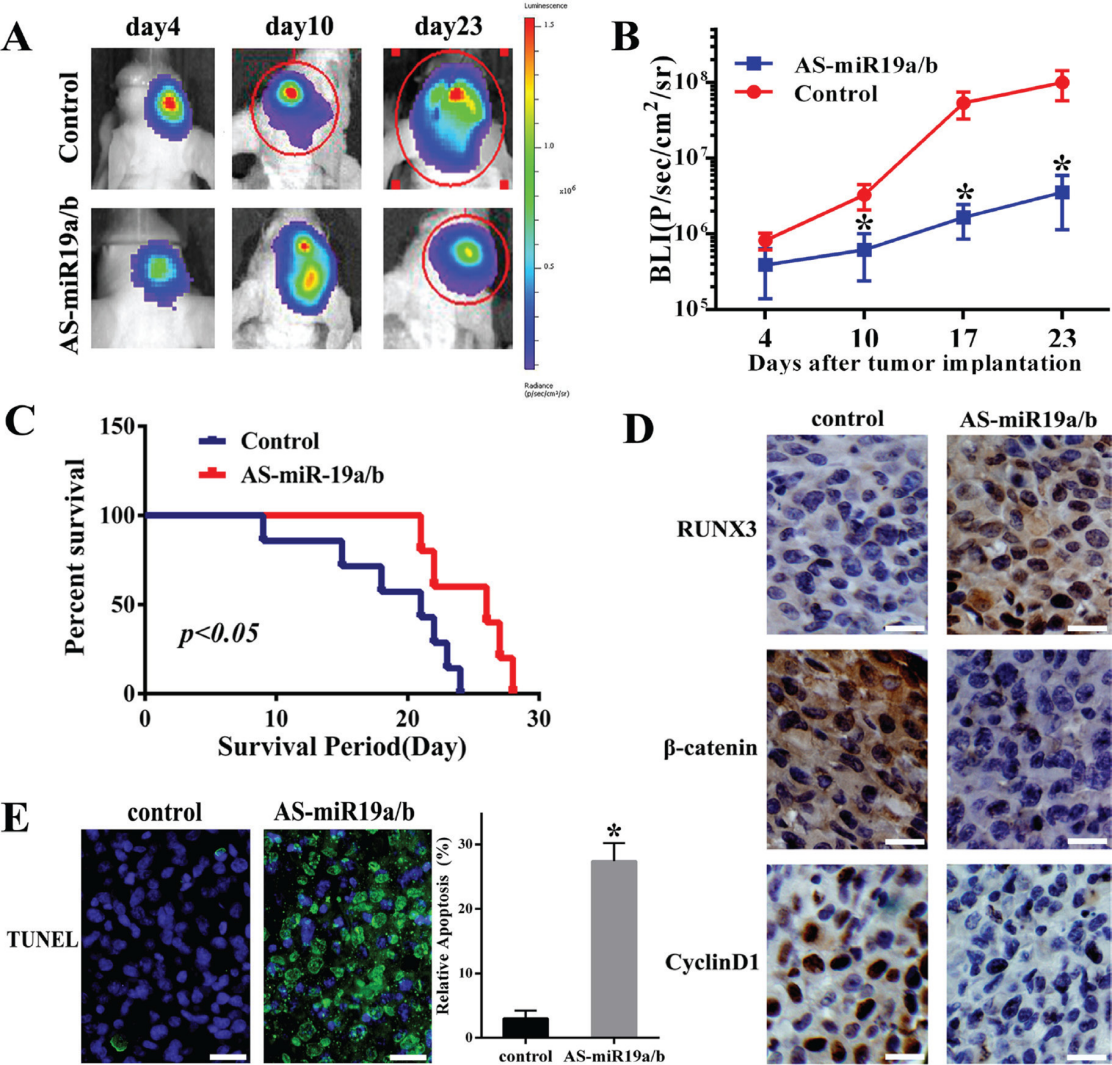
Knockdown of miR-19a/b expression suppresses U87 tumor growth *in vivo* (**A**) U87 cells pretreated with a lentivirus containing luciferase reporter or cotransfection of miR-19a/b inhibitors were implanted in the right forebrain of nude mice, and tumorigenicity was analysized by bioluminescence imaging. Tumor formation assessed by bioluminescent signal were examined at day 4, 10, 23 after tumor implantation. (**B**) Tumor growth curves were analyzed. The data are described as mean ± SD. ^*^p < 0.05. (**C**) Overall survival was determined by Kaplan-Meier analysis, and log-rank test was used to evaluate the statistical of the differences. (**D**) At the end of experiment, Immunohistochemistry was used to assess the expression of RUNX3, β-catenin and CyclinD1 in tumor sections between control and AS-miR-19a/b group. (**E**) TUNEL analysis on intracranical tumor sections indicating more apoptotic cells compared with control group. ^*^p < 0.05.

AS-miR-19a/b repressed the proliferation, invasion of glioma cells and targeted regulation for RUNX3 *in vitro*, we further examined its effect on tumor growth *in vivo*. Bioluminescence imaging was used to assess the tumor formation when the nude mice were intracranial transplanted with U87 cells that stably express luciferase and miR-19a/b inhibitors. As-miR19a/b-treated U87 cells displayed a markedly reduction of the tumor (Figure 6A, 6B). To analyze the survival times between two groups, we then generated Kaplan-Meier survival curves, which demonstrated that AS-miR19a/b significantly prolonged survival (Figure 6C). Moreover, AS-miR19a/b group exhibited increased expression of RUNX3 and decreased expression of β-catenin and CyclinD1 as verified by IHC assay (Figure 6D). Furthermore, AS-miR19a/b group had more apoptotic nucleuses verified by TUNEL evaluation (Figure 6E).

## DISCUSSION

Our previous study has verified that miR-19a/b was over-expressed in glioma cell lines and tissue specimens with the distinct grade (Pathol. Oncol. Res. (2013) 19:847–853). Meanwhile, we also demonstrated that RUNX3 was down-regulated in GBM cell lines and glioma tissues (data not shown). Based on our data studies for tissue chip, in the present study, we first display the relationship between the expression of miR-19a/b and RUNX3 with WHO grade respectively, suggesting the inverse correlation both miR-19a/b and RUNX3. Furthermore, we analyzed 43 resected tissue specimens verified by Real-time PCR and IHC, also indicating that RUNX3 had reduced expression in tissue specimens and negatively correlated with the grade. Our data demonstrated that miR-19 and RUNX3 are considered as oncogene factor and tumor suppressor in glioma respectively, however, the functional roles of them as well as the underlying relationship between them remain poorly understood.

MicroRNAs are increasingly shown as oncogene or anti-tumor genes that regulate gene expression in post transcription [14, 15]. Recent study demonstrated that microRNA-19a promotes gliomas cell growth by repressing LRIG1 which is a pan-negative regulator of membrane-bound receptor tyrosine kinases(RTKs) [16]. Our previous data also verified that PTEN is a candicate direct target gene by miR-19a/b, which is consistent with the results for breast cancer and T-cell lymphoma [10, 17, 18]. Using four algorithms (TargetScan, PicTar, miRanda, miRbase Target), we were prone to predict that miR-19a/b directly binds to 3′UTR of RUNX3. Furthermore, we performed a 3′UTR luciferase assay and observed that luciferase activity was remarkably increased after co-transfection of the miR-19a/b inhibitors and a 3′UTR vector containing target sequence.RUNX3 protein level was also markedly up-regulated in LN229 and U87 cells that were transfected with the miR-19a/b inhibitors, suggesting that RUNX3 is a direct target of miR-19a/b.

RUNX3 has been identified as a tumour suppressor gene in a variety of solid tumours including gastric carcinoma, breast carcinoma, hepatocellular cancer and oral squamous-cell carcinoma [19–22]. Mueller *et al.* has reported that down-regulation of RUNX3 by hypermethylation in glioblastoma [12]. There were relevant reports with miRNA medicated inactivation of RUNX3 such as miR-532-5p in gastric cancer and miR-4295 in gliomas [23, 24]. Our data demonstrated that miR-19a/b medicated regulation for RUNX3 is another potential mechanism for its inactivation.

In our study, we, for the first time, comprehensively analyze the role of miR-19a/b inhibition for gliomas malignant phenotype. Our results demonstrated that low expression of miR-19a/b inhibited cell proliferation, invasion and induced the cell cycle G0/G1 arrest. Cell apoptosis was also dramatically increased after miR-19a/b suppression. Additionally, silencing of RUNX3 could practically reverse the suppressive effect of miR-19a/b inhibitors. Up-regulation of miR-19a/b also could partly abrogate the inhibitory effect of RUNX3. Our *in vivo* tumorigenic experiment study implicated that suppression of miR19a/b was therapeutically beneficial. These results suggest that inhibition of miR-19a/b could repress the growth of glioma cell *in vitro* and *in vivo*, at least partly though up-regulation of RUNX3.

It has been reported that RUNX3 forms a ternary complex with β-catenin/TCF4 and attenuates Wnt signaling activity in intestinal tumorigenesis [25]. By using TOP/FOP flash luciferase assay, we determined that knockdown of miR-19a/b or restoration of RUNX3 all significantly repressed the β-catenin/TCF4 transcription activities. In addition, multiple downstream targets of the β-catenin/TCF4 pathway, such as cyclinD1, c-MYC, VEGF and AKT1 were all reduced in cells with the knockdown of miR-19a/b. Thus, these data support the conclusion that miR-19a/b regulates survival of glioma cells via modulating RUNX3 inhibition of the β-catenin/TCF4 transcription.

In summary, our study provides new insights into the role of miR-19a/b in human glioma and confirms that miR-19 is able to inhibit RUNX3 expression level through directly binding to the 3′UTR region of RUNX3 which medicates the suppression of β-catenin/TCF4 transcription. Based on the regulation of multiple targets as well as signaling pathways, inhibition of miR-19a/b expression may represent a novel therapeutic target in glioblastoma.

## MATERIALS AND METHODS

### Tissue samples and clinical data

Tissue microarray for detecting miR-19a/b expression was prepared by ChaoYing Biotechnology (Shanxi, China) and the concrete informations were described in the published paper [10]. A tissue microarray for RUNX3 expression contained 59 glioma samples with different grades, including 9 grade I samples, 20 grade II samples, 17 grade III samples and 13 grade IV samples (Table 1). Forty-three freshly resected astrocytic glioma samples were collected with patient’s consent at the time of operation and classified according to 2007 WHO categories. Samples included 12 WHO grade I-II tumors, 15 WHO grade III tumors and 16 WHO grade IV tumors (Table 2). Freshly resected tissue was immediately frozen at liquid nitrogen for subsequent total RNA extraction. Five normal adult brain tissue specimens were obtained with informed consent from patients undergoing post-trauma surgery for severe traumatic brain injury (TBI).

**Table 1:** Tissue microarray pathology and clinical classification for 59 glioma samples

No.	Age	Sex	Pathology (WHO)	No.	Age	Sex	Pathology (WHO)
01	47	M	Astrocytoma I	31	59	M	Glioblastoma III
02	35	M	Astrocytoma I	32	64	F	Glioblastoma III
03	19	F	Astrocytoma I	33	41	F	Glioblastoma III
04	21	M	Astrocytoma I	34	40	F	Glioblastoma III
05	12	M	Astrocytoma I	35	49	M	Glioblastoma III
06	41	F	Astrocytoma I	36	18	F	Glioblastoma III
07	32	F	Astrocytoma I	37	35	F	Glioblastoma III
08	42	M	Astrocytoma I	38	43	M	Glioblastoma III
09	51	F	Astrocytoma II	39	33	M	Glioblastoma III
10	52	M	Astrocytoma II	40	67	M	Glioblastoma III
11	40	F	Astrocytoma II	41	42	M	Glioblastoma III
12	50	M	Astrocytoma II	42	59	M	Glioblastoma III
13	37	M	Astrocytoma II	43	33	M	Glioblastoma III
14	51	M	Astrocytoma II	44	65	F	GBM IV
15	42	F	Astrocytoma II	45	54	M	Glioblastoma III
16	27	F	Astrocytoma II	46	63	M	GBM IV
17	56	M	Astrocytoma II	47	39	M	GBM IV
18	57	F	Astrocytoma II	48	9	M	GBM IV
19	53	M	Astrocytoma I	49	37	M	GBM IV
20	50	M	Astrocytoma II	50	34	M	GBM IV
21	71	M	Astrocytoma II	51	47	F	Glioblastoma III
22	45	F	Astrocytoma II	52	33	F	GBM IV
23	37	F	Astrocytoma II	53	61	F	GBM IV
24	40	F	Astrocytoma II	54	76	M	Glioblastoma III
25	66	M	Astrocytoma II	55	40	F	GBM IV
26	46	M	Astrocytoma II	56	23	M	GBM IV
27	49	F	Astrocytoma II	57	64	M	GBM IV
28	49	M	Astrocytoma III	58	22	M	GBM IV
29	10	M	Astrocytoma III	59	43	M	GBM IV
30	32	M	Astrocytoma II				

**Table 2:** Clinicopathologic parameters of 43 glioma samples

No.	Age	Sex	Pathogy (WHO)	No.	Age	Sex	Pathogy (WHO)
01	36	M	Astrocytoma I	23	30	M	Astrocytoma III
02	48	F	Astrocytoma I	24	47	M	Oligodendroglioma III
03	35	M	Astrocytoma I	25	27	M	Astrocytoma III
04	8	M	Astrocytoma II	26	9	F	Oligodendroglioma III
05	17	F	Astrocytoma II	27	54	M	Astrocytoma III
06	3	M	Astrocytoma II	28	31	M	GBM IV
07	23	M	Astrocytoma II	29	2	F	Astrocytoma IV
08	33	F	Astrocytoma II	30	6	F	Astrocytoma IV
09	12	M	Oligodendroglioma II	31	24	M	Astrocytoma IV
10	7	M	Oligodendroglioma II	32	51	M	GBM IV
11	10	M	Oligodendroglioma II	33	32	F	GBM IV
12	42	M	Oligodendroglioma II	34	22	F	GBM IV
13	5	M	Astrocytoma III	35	25	M	GBM IV
14	49	F	Astrocytoma III	36	20	F	GBM IV
15	4	M	Astrocytoma III	37	44	F	GBM IV
16	50	F	Oligodendroglioma III	38	46	F	GBM IV
17	13	M	Astrocytoma III	39	26	M	GBM IV
18	53	M	Astrocytoma III	40	40	F	GBM IV
19	41	M	Astrocytoma III	41	29	M	GBM IV
20	18	M	Astrocytoma III	42	16	M	GBM IV
21	38	M	Astrocytoma III	43	21	M	GBM IV
22	14	M	Astrocytoma III				

### Cell culture and transfection

Human glioma cells (U87 and LN229) were obtained from the China Academia Sinica Cell Repository, Shanghai, China and were cultured in Dulbecco’s modified Eagle medium (DMEM) supplemented with 10% heat-inactivated fetal bovine serum (FBS, Hyclone). The cells were maintained in a humidified atmosphere at 5% CO2 atmosphere at 37°C. The cells were transfected using Lipofectamine 2000 (Invitrogen, Carlsbad, USA) according to the manufacturer’s instructions.

### Oligonucleotides and adenovirus infection

The Has-miR-19a/b inhibitor and mimics were chemically synthesized and purified by high-performance liquid chromatography (GenePharma, Shanghai, China). The miR-19a inhibitor was 5′-UCAGUUUUGCAUAGAUUUGCACA-3′. The miR-19b inhibitor was 5′-UCAGUUUUGCAUGGAUUUGCACA -3′. Adenovirus containing a RUNX3 cDNA (rAd-RUNX3) was obtained from Genesil (Wuhan, China). We used RUNX3 siRNA (Cell Signaling Technology, USA) to knock down the expression of RUNX3.

### Real-time PCR

Total RNA from tissues and cells was isolated using TRIzol reagent (Invitrogen) for mRNA analyses. qRT-PCR reactions were performed with TaqMan reverse transcription reagents and SYBR Green PCR Master Mix (Applied Biosystems) according to the manual. Normalization was performed on the U6 microRNA levels. qRT-PCR for miRNA detection was performed with TaqMan miRNA assays (Applied Biosystems) and GAPDH (Life Technologies, Carlsbad, CA, USA) was used as RUNX3 mRNA internal control.

### Proliferation assay

U87 and LN229 cells were seeded into 96-well plates at 4000 cells per well respectively. After transfection as described previously, on each day of consecutive 7 days, 20 mL 3-4,5-dimethyl-2-thiazolyl)-2,5-diphenyl-2-H-tetrazolium bromide (MTT) (5 mg/mL) was added to each well and the cells were incubated at 37 uC for additional 4 h. and the supernatant was discarded. All proliferation assays were repeated as independent experiments at least twice.

### Cell-cycle analysis

U87 and LN229 cells (1 × 10^5^ cells) were plated in 60 mm culture plates, After 2 days, the cells were trypsinized, fixed in 75% ethanol, washed with PBS, and then labeled with propidium iodide (Sigma-Aldrich) in the presence of RNase A (Sigma-Aldrich) for 30 min. Samples were run on a FACScan flow cytometer (Becton-Dickinson, FL, NJ, USA), and the percentages of cells within each phase of the cell cycle were analyzed using Modifit software.

### *In vitro* invasion assays

Transwell membranes coated with Matrigel (BD Biosciences, San Jose, CA) were used to analysize the invasive activity of GBM cells. (1 × 10^5^) in 100 μl of serum free DMEM were added into the upper compartment of the chamber and 200 μl conditioned medium fromU87 and LN229 was used as chemoattractant and placed in bottom chamber. After 24 hour incubation at 37°C, the medium was removed from the upper chamber. The noninvaded cells on the upper surface of the inserted filter were scraped off with a cotton swab. The cells that had migrated into the lower surface of the inserted filter were fixed with methanol. The number of cells invading through the matrigel was counted using three randomLy selected visual fields.

### Luciferase reporter assay

A fragment of the human RUNX3 3′UTR containing a putative miR-19a/b binding site was cloned into a dual-luciferase plasmid vector (pEZX-MT01). The cells were lysed 48 h after transfection, and the ratio of firefly luciferase activity to Renilla luciferase activity was measured with a dual-luciferase assay (GeneCopoeia, USA).

To evaluate the β-catenin/Tcf-4 transcriptional activity, we used the TOP-FLASH and FOP-FLASH (Upstate) luciferase reporter constructs. TOP-FLASH (with 3 repeats of the Tcf-binding site) or FOP-FLASH (with 3 repeats of a mutated Tcf-binding site) plasmids were transfected into cells treated with Ad-RUNX3. After 48 h incubation, luciferase activity was measured using a dualluciferase reporter system (Promega). Luciferase activity was measured 48 h after transfection with the Dual-luciferase reporter assay system. The Renilla luciferase activity was used as an internal control.

### Apoptosis assays

Apoptosis was quantified 48 h after transfection, using annexin V labeling. For the annexin V assay, an annexin V-FITC–labeled Apoptosis Detection Kit (Abcam) was used according to the manufacturer’s protocol and the TUNEL assay was used to detect apoptosis in tumor specimens [26].

### Western blot and immunohistochemical staining

Western blot and IHC assays were performed as previously described. IHC scores were performed using a semiquantitative 5-category grading system [27, 28].

### Intracranial nude mouse models

BALB/c-A nude mice at 4 weeks of age were purchased from the Animal Center at the Cancer Institute at Chinese Academy of Medical Science. To establish intracranial gliomas, 5 × 10^5^ U87 glioblastoma cells pretreated with AS-miR-19a/b or vector were implanted stereotactically [29]. Bioluminescence imaging was used to detect intracranial tumor growth using the IVIS Imaging System (caliper Life Sciences) [30].

### Statistical analysis

Statistics were performed using the SPSS Graduate Pack, version 16.0, statistical software (SPSS). Descriptive statistics, including mean and SE and 1-way analysis of variance, were used to determine statistically significant differences. Overall survival curves were plotted according to the Kaplan-Meier method. *P* < 0.05 was considered to be statistically significant.
